# 1-{2-[4-(4-Nitro­phen­yl)piperazin-1-yl]eth­yl}-4-aza-1-azoniabicyclo­[2.2.2]octane iodide

**DOI:** 10.1107/S1600536812024531

**Published:** 2012-06-02

**Authors:** Anssi Peuronen, Manu Lahtinen

**Affiliations:** a University of Jyväskylä, Department of Chemistry, PO Box 35, FIN-40014 JY, Finland

## Abstract

The title compound, C_18_H_28_N_5_O_2_^+^·I^−^, was observed as a main product in an intended 1:1 reaction between 4-iodo­nitro­benzene and 1,4-diaza­bicyclo­[2.2.2]octane (DABCO). In the reaction, DABCO undergoes a ring opening to yield a quaternary salt of DABCO and 1-ethyl-4-(4-nitro­phen­yl)piperazine with an iodide anion. The crystal structure determination was carried out as no crystal structure had been previously reported in the investigations describing the corresponding reaction with 4-chloro­nitro­benze. Indeed, the crystal structure of the title compound confirms the mol­ecular composition proposed earlier for the analogous chloride salt. The cation conformation is similar to the previously reported dinitro analogue 1-{2-[4-(2,4-dinitro­phen­yl)piperazin-1-yl]eth­yl}-4-aza-1-azoniabicyclo­[2.2.2]octane chloride [Clegg *et al.* (2004 ▶). *Acta Cryst.* E**60**, o291–o293]. The crystal packing is dominated by cation⋯I^−^ inter­actions in addition to weak inter­molecular C—H⋯O_2_N and C—H⋯N inter­actions between the cations.

## Related literature

For a possible route of synthesis for the chloride salt of the title compound, see: Ross & Finkelstein (1963 ▶). For a related structure, see: Clegg *et al.* (2004 ▶). For the synthesis of the intended 1:1 product of DABCO and 4-iodo­nitro­benzene, see Ibata *et al.* (1987 ▶).
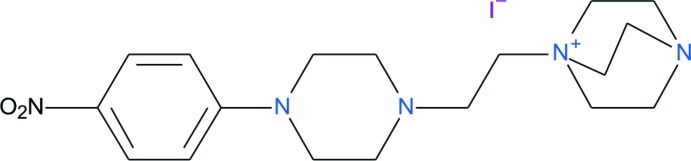


## Experimental

### 

#### Crystal data

C_18_H_28_N_5_O_2_^+^·I^−^
*M* *_r_* = 473.35
Monoclinic, 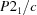

*a* = 9.758 (1) Å
*b* = 10.702 (1) Å
*c* = 20.187 (2) Å
β = 110.124 (3)°
*V* = 1979.4 (3) Å^3^
*Z* = 4
Mo *K*α radiation
μ = 1.64 mm^−1^
*T* = 123 K
0.40 × 0.24 × 0.16 mm

#### Data collection

Bruker–Nonius KappaCCD diffractometer with an APEXII detector
Absorption correction: multi-scan (*SADABS*; Sheldrick, 2008*b* ▶) *T*_min_ = 0.617, *T*_max_ = 0.746
12011 measured reflections
3855 independent reflections
3473 reflections with *I* > 2σ(*I*)
*R*_int_ = 0.032

#### Refinement

*R*[*F* ^2^ > 2σ(*F* ^2^)] = 0.029
*wR*(*F* ^2^) = 0.060
*S* = 1.10
3855 reflections
235 parameters
H-atom parameters constrained
Δρ_max_ = 0.48 e Å^−3^
Δρ_min_ = −0.44 e Å^−3^

### 

Data collection: *COLLECT* (Bruker, 2008 ▶); cell refinement: *DENZO-SMN* (Otwinowski & Minor, 1997 ▶); data reduction: *DENZO-SMN*; program(s) used to solve structure: *SHELXS97* (Sheldrick, 2008*a* ▶); program(s) used to refine structure: *SHELXL97* (Sheldrick, 2008*a* ▶); molecular graphics: *Mercury* (Macrae *et al.* 2008 ▶); software used to prepare material for publication: *SHELXL97*.

## Supplementary Material

Crystal structure: contains datablock(s) I, global. DOI: 10.1107/S1600536812024531/nr2029sup1.cif

Structure factors: contains datablock(s) I. DOI: 10.1107/S1600536812024531/nr2029Isup2.hkl

Supplementary material file. DOI: 10.1107/S1600536812024531/nr2029Isup3.cml

Additional supplementary materials:  crystallographic information; 3D view; checkCIF report

## Figures and Tables

**Table 1:** Hydrogen-bond geometry (Å, °)

*D*—H⋯*A*	*D*—H	H⋯*A*	*D*⋯*A*	*D*—H⋯*A*
C2—H2*B*⋯O1^i^	0.99	2.52	3.051 (3)	113
C5—H5*A*⋯O2^ii^	0.99	2.47	3.414 (3)	160
C6—H6*B*⋯O1^ii^	0.99	2.59	3.141 (3)	116
C14—H14⋯I1^i^	0.95	3.02	3.914 (3)	158
C17—H17⋯O2^iii^	0.95	2.48	3.412 (3)	168
C18—H18⋯N1^iv^	0.95	2.45	3.384 (3)	166

Symmetry codes: (i) 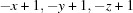; (ii) 
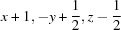
; (iii) 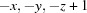; (iv) 
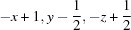
.

**Fig. 1.**
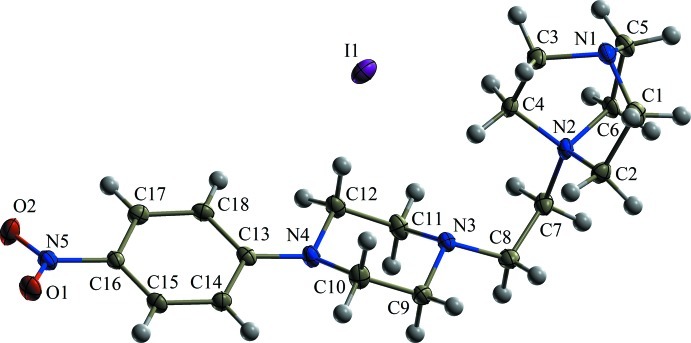
Asymmetric unit and labeling scheme of the title compound. Ellipsoids are presented at the 50% probability level.

**Fig. 2.**
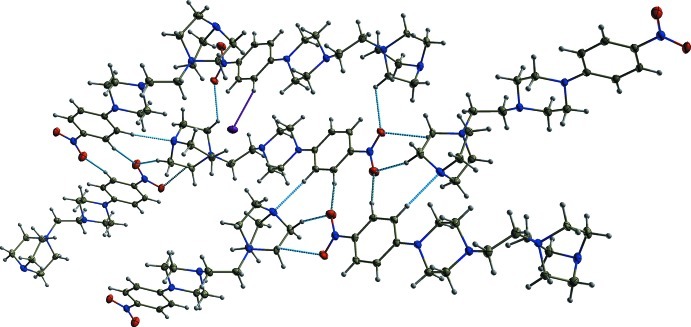
Anion-cation and cation-cation interactions viewed along the crystallographic a-axis. Ellipsoids are presented at the 50% probability level.

**Fig. 3.**
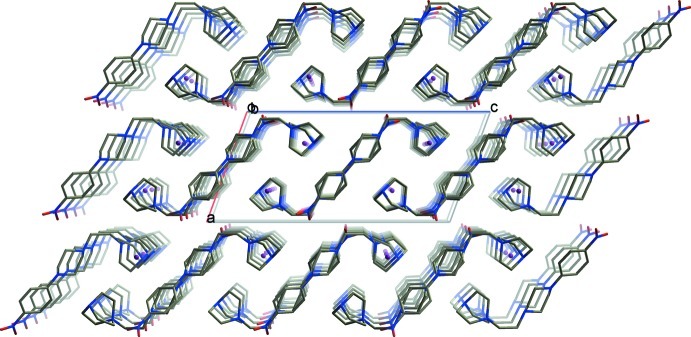
Packing of the ion pairs viewed along the crystallographic b-axis. The hydrogen atoms have been omitted for clarity.

## supplementary crystallographic information

### Comment

The reaction between DABCO and 4-chloronitrobenzene has first been reported by Ross & Finkelstein (1963). The product obtained was identified as the chloride salt analogue of the title compound, 1-(4-nitrophenyl)-4-aza-1-azoniabicyclo[2.2.2]octane chloride, instead of the expected 1:1 product. We were interested in the synthesis of the elusive 1:1 product for its potential applications in supramolecular chemistry based on halogen bonding interactions. We tried to use 4-iodonitrobenzene in order to obtain the 1:1 product. Regardless of the milder conditions (THF at reflux for 48 h) and a different halobenzene, the corresponding reaction proceeds according to the aforementioned route yielding an analogous iodide salt, 1-(4-nitrophenyl)-4-aza-1-azoniabicyclo[2.2.2]octane iodide, to the compound described above. Nonetheless, despite the failure in the intended synthesis, the crystal structure of the title compound provides a crystallographic evidence for the previously described reaction between DABCO and a 4-halonitrobenzene. Furthermore, our investigation suggests that changing the halogen atom in the 4-halonitrobenzene from chloride to iodine has evidently no effect in the outcome of the reaction (except that of different anion). A possible route for the anticipated 1:1 product is described by Ibata *et al.* (1987).

The cation of the title salt lies in a conformation similar to the previously reported dinitrobenzene analogue (Clegg *et al.*, 2004). The labeling scheme is shown in Fig. 1. The intermolecular interactions in the crystal structure comprise mostly of weak C—H···O_2_N interactions between DABCO –CH_2_ groups and the –NO_2_ groups [C···O distances range from 3.051 (3) to 3.414 (3)]. These short intermolecular contacts are most likely due to the attractive interactions between highly electronegative O atoms in the nitro groups and electropositive H atoms near the quaternary ammonium center. These are accompanied by presumably weaker aryl –CH···O_2_N [*d*(C17···O2) = 3.412 (3)], aryl –CH···I^-^ [*d*(C14···I1) = 3.914 (3)] and aryl –CH···N (DABCO) [*d*(C18···N1) = 3.412 (3)] interactions (Fig. 2). The ordering of the ion pairs are shown in Fig. 3.

### Experimental

The title compound was obtained as a major product in a reaction between DABCO (2.0 mmol) and 4-iodonitrobenzene (2.0 mmol) carried out in THF (48 h at reflux). After the removal of solvent the yellow oily residue was precipitated with dichloromethane, filtered and recrystallized from water/acetone mixture to yield a batch of yellow crystals of the title compound.

### Refinement

All H atoms were refined as riding atoms with fixed isotropic displacement parameters 1.2 times larger than corresponding host carbon atoms. C—H distances were refined as 0.95 Å for aromatic and 0.99 Å for methylene H atoms. All non-hydrogen atoms were refined anisotropically.

### Crystal data

**Table:**

C_18_H_28_N_5_O_2_^+^·I^−^	*F*(000) = 960
*M**_r_* = 473.35	*D*_x_ = 1.588 Mg m^−^^3^
Monoclinic, *P*2_1_/*c*	Mo *K*α radiation, λ = 0.71069 Å
Hall symbol: -P 2ybc	Cell parameters from 489 reflections
*a* = 9.758 (1) Å	θ = 1.0–26.0°
*b* = 10.702 (1) Å	µ = 1.64 mm^−^^1^
*c* = 20.187 (2) Å	*T* = 123 K
β = 110.124 (3)°	Block, yellow
*V* = 1979.4 (3) Å^3^	0.40 × 0.24 × 0.16 mm
*Z* = 4	

### Data collection

**Table:**

Bruker–Nonius KappaCCD diffractometer with ApexII detector	3855 independent reflections
Radiation source: fine-focus sealed tube	3473 reflections with *I* > 2σ(*I*)
Graphite monochromator	*R*_int_ = 0.032
Detector resolution: 9 pixels mm^-1^	θ_max_ = 26.0°, θ_min_ = 2.2°
φ and ω scans	*h* = −12→10
Absorption correction: multi-scan (*SADABS*; Sheldrick, 2008*b*)	*k* = −12→13
*T*_min_ = 0.617, *T*_max_ = 0.746	*l* = −24→24
12011 measured reflections	

### Refinement

**Table:**

Refinement on *F*^2^	Primary atom site location: structure-invariant direct methods
Least-squares matrix: full	Secondary atom site location: difference Fourier map
*R*[*F*^2^ > 2σ(*F*^2^)] = 0.029	Hydrogen site location: inferred from neighbouring sites
*wR*(*F*^2^) = 0.060	H-atom parameters constrained
*S* = 1.10	*w* = 1/[σ^2^(*F*_o_^2^) + (0.*P*)^2^ + 2.7637*P*] where *P* = (*F*_o_^2^ + 2*F*_c_^2^)/3
3855 reflections	(Δ/σ)_max_ = 0.001
235 parameters	Δρ_max_ = 0.48 e Å^−^^3^
0 restraints	Δρ_min_ = −0.44 e Å^−^^3^

### Special details

**Table:**

Geometry. All s.u.'s (except the s.u. in the dihedral angle between two l.s. planes) are estimated using the full covariance matrix. The cell s.u.'s are taken into account individually in the estimation of s.u.'s in distances, angles and torsion angles; correlations between s.u.'s in cell parameters are only used when they are defined by crystal symmetry. An approximate (isotropic) treatment of cell s.u.'s is used for estimating s.u.'s involving l.s. planes.
Refinement. Refinement of *F*^2^ against ALL reflections. The weighted *R*-factor *wR* and goodness of fit *S* are based on *F*^2^, conventional *R*-factors *R* are based on *F*, with *F* set to zero for negative *F*^2^. The threshold expression of *F*^2^ > 2σ(*F*^2^) is used only for calculating *R*-factors(gt) *etc*. and is not relevant to the choice of reflections for refinement. *R*-factors based on *F*^2^ are statistically about twice as large as those based on *F*, and *R*- factors based on ALL data will be even larger.

### Fractional atomic coordinates and isotropic or equivalent isotropic displacement parameters (Å^2^)

**Table:**

	*x*	*y*	*z*	*U*_iso_*/*U*_eq_	
C1	0.8248 (3)	0.5722 (3)	0.22628 (15)	0.0253 (6)	
H1A	0.7838	0.6532	0.2341	0.030*	
H1B	0.9068	0.5895	0.2094	0.030*	
C2	0.8816 (3)	0.4998 (2)	0.29637 (14)	0.0191 (6)	
H2A	0.9891	0.5078	0.3174	0.023*	
H2B	0.8381	0.5339	0.3302	0.023*	
C3	0.6055 (3)	0.4544 (3)	0.20373 (14)	0.0212 (6)	
H3A	0.5201	0.4184	0.1663	0.025*	
H3B	0.5708	0.5250	0.2255	0.025*	
C4	0.6750 (3)	0.3538 (2)	0.26039 (14)	0.0180 (6)	
H4A	0.6454	0.3673	0.3021	0.022*	
H4B	0.6423	0.2695	0.2412	0.022*	
C5	0.7831 (3)	0.3924 (3)	0.15276 (14)	0.0234 (6)	
H5A	0.8425	0.4213	0.1247	0.028*	
H5B	0.7079	0.3343	0.1232	0.028*	
C6	0.8814 (3)	0.3235 (2)	0.21871 (13)	0.0178 (6)	
H6A	0.8682	0.2321	0.2119	0.021*	
H6B	0.9851	0.3436	0.2273	0.021*	
C7	0.9208 (3)	0.2816 (2)	0.34290 (14)	0.0199 (6)	
H7A	1.0238	0.2759	0.3455	0.024*	
H7B	0.8787	0.1965	0.3332	0.024*	
C8	0.9188 (3)	0.3226 (3)	0.41463 (14)	0.0215 (6)	
H8A	0.9851	0.2675	0.4511	0.026*	
H8B	0.9583	0.4086	0.4239	0.026*	
C9	0.7828 (3)	0.3793 (2)	0.48911 (14)	0.0210 (6)	
H9A	0.8214	0.4653	0.4912	0.025*	
H9B	0.8502	0.3313	0.5290	0.025*	
C10	0.6337 (3)	0.3833 (2)	0.49568 (15)	0.0218 (6)	
H10A	0.6416	0.4200	0.5419	0.026*	
H10B	0.5692	0.4378	0.4582	0.026*	
C11	0.7208 (3)	0.1927 (2)	0.42183 (15)	0.0219 (6)	
H11A	0.7876	0.1456	0.4623	0.026*	
H11B	0.7186	0.1503	0.3779	0.026*	
C12	0.5692 (3)	0.1928 (2)	0.42620 (14)	0.0205 (6)	
H12A	0.5006	0.2341	0.3839	0.025*	
H12B	0.5361	0.1056	0.4272	0.025*	
C13	0.4545 (3)	0.2381 (2)	0.51467 (13)	0.0163 (5)	
C14	0.4455 (3)	0.3041 (2)	0.57372 (13)	0.0172 (5)	
H14	0.5165	0.3662	0.5954	0.021*	
C15	0.3362 (3)	0.2803 (2)	0.60037 (14)	0.0171 (5)	
H15	0.3308	0.3265	0.6396	0.021*	
C16	0.2337 (3)	0.1884 (2)	0.56971 (13)	0.0158 (5)	
C17	0.2390 (3)	0.1219 (2)	0.51154 (13)	0.0176 (6)	
H17	0.1682	0.0591	0.4910	0.021*	
C18	0.3463 (3)	0.1465 (2)	0.48361 (14)	0.0179 (6)	
H18	0.3481	0.1019	0.4432	0.021*	
N1	0.7115 (2)	0.5005 (2)	0.17234 (12)	0.0215 (5)	
N2	0.8396 (2)	0.36461 (18)	0.28111 (11)	0.0152 (4)	
N3	0.7755 (2)	0.32088 (19)	0.42256 (11)	0.0167 (5)	
N4	0.5684 (2)	0.25860 (19)	0.48977 (11)	0.0174 (5)	
N5	0.1165 (2)	0.1644 (2)	0.59637 (11)	0.0189 (5)	
O1	0.0900 (2)	0.24296 (17)	0.63502 (10)	0.0241 (4)	
O2	0.0460 (2)	0.06682 (18)	0.57861 (11)	0.0287 (5)	
I1	0.289527 (19)	0.471091 (16)	0.287677 (10)	0.02555 (7)	

### Atomic displacement parameters (Å^2^)

**Table:**

	*U*^11^	*U*^22^	*U*^33^	*U*^12^	*U*^13^	*U*^23^
C1	0.0279 (16)	0.0193 (13)	0.0288 (16)	−0.0030 (12)	0.0102 (13)	0.0036 (12)
C2	0.0184 (14)	0.0169 (12)	0.0216 (15)	−0.0027 (11)	0.0065 (12)	−0.0036 (11)
C3	0.0163 (14)	0.0280 (14)	0.0207 (15)	0.0031 (11)	0.0081 (12)	0.0045 (11)
C4	0.0124 (13)	0.0227 (13)	0.0201 (14)	−0.0022 (10)	0.0069 (11)	0.0016 (11)
C5	0.0238 (15)	0.0323 (15)	0.0174 (14)	−0.0008 (12)	0.0115 (12)	−0.0004 (12)
C6	0.0195 (14)	0.0218 (13)	0.0170 (14)	−0.0006 (11)	0.0123 (12)	−0.0034 (10)
C7	0.0156 (14)	0.0217 (13)	0.0235 (15)	0.0046 (11)	0.0083 (12)	0.0063 (11)
C8	0.0169 (14)	0.0284 (15)	0.0185 (14)	0.0022 (11)	0.0051 (12)	0.0038 (11)
C9	0.0214 (15)	0.0242 (14)	0.0178 (14)	−0.0051 (11)	0.0075 (12)	−0.0018 (11)
C10	0.0261 (15)	0.0172 (13)	0.0259 (15)	−0.0026 (11)	0.0140 (13)	−0.0040 (11)
C11	0.0282 (16)	0.0191 (13)	0.0235 (15)	0.0021 (11)	0.0154 (13)	0.0010 (11)
C12	0.0274 (16)	0.0176 (13)	0.0200 (15)	−0.0039 (11)	0.0127 (12)	−0.0039 (11)
C13	0.0186 (14)	0.0162 (12)	0.0152 (13)	0.0039 (10)	0.0073 (11)	0.0023 (10)
C14	0.0188 (14)	0.0158 (12)	0.0173 (14)	−0.0018 (10)	0.0067 (11)	−0.0033 (10)
C15	0.0200 (14)	0.0176 (13)	0.0160 (13)	0.0039 (11)	0.0091 (11)	−0.0024 (10)
C16	0.0147 (13)	0.0190 (13)	0.0147 (13)	0.0019 (10)	0.0062 (11)	0.0025 (10)
C17	0.0206 (14)	0.0135 (12)	0.0187 (14)	−0.0003 (10)	0.0066 (12)	−0.0018 (10)
C18	0.0210 (14)	0.0177 (13)	0.0147 (13)	0.0019 (11)	0.0060 (11)	−0.0029 (10)
N1	0.0206 (12)	0.0265 (12)	0.0198 (13)	0.0000 (10)	0.0101 (10)	0.0049 (10)
N2	0.0148 (11)	0.0148 (10)	0.0190 (12)	0.0001 (8)	0.0098 (9)	−0.0011 (9)
N3	0.0164 (12)	0.0188 (11)	0.0164 (12)	−0.0005 (9)	0.0076 (9)	−0.0003 (9)
N4	0.0216 (12)	0.0156 (10)	0.0184 (12)	−0.0019 (9)	0.0115 (10)	−0.0033 (9)
N5	0.0190 (12)	0.0227 (12)	0.0166 (12)	0.0017 (9)	0.0080 (10)	0.0004 (9)
O1	0.0269 (11)	0.0262 (10)	0.0244 (11)	0.0014 (8)	0.0154 (9)	−0.0056 (8)
O2	0.0246 (11)	0.0314 (11)	0.0348 (12)	−0.0116 (9)	0.0161 (10)	−0.0109 (9)
I1	0.01937 (11)	0.02237 (11)	0.03199 (13)	−0.00198 (7)	0.00509 (8)	−0.00562 (7)

### Geometric parameters (Å, º)

**Table:**

C1—N1	1.472 (4)	C9—N3	1.461 (3)
C1—C2	1.539 (4)	C9—C10	1.506 (4)
C1—H1A	0.9900	C9—H9A	0.9900
C1—H1B	0.9900	C9—H9B	0.9900
C2—N2	1.507 (3)	C10—N4	1.466 (3)
C2—H2A	0.9900	C10—H10A	0.9900
C2—H2B	0.9900	C10—H10B	0.9900
C3—N1	1.472 (3)	C11—N3	1.470 (3)
C3—C4	1.547 (4)	C11—C12	1.511 (4)
C3—H3A	0.9900	C11—H11A	0.9900
C3—H3B	0.9900	C11—H11B	0.9900
C4—N2	1.519 (3)	C12—N4	1.466 (3)
C4—H4A	0.9900	C12—H12A	0.9900
C4—H4B	0.9900	C12—H12B	0.9900
C5—N1	1.474 (3)	C13—N4	1.386 (3)
C5—C6	1.535 (4)	C13—C14	1.414 (3)
C5—H5A	0.9900	C13—C18	1.418 (4)
C5—H5B	0.9900	C14—C15	1.374 (4)
C6—N2	1.517 (3)	C14—H14	0.9500
C6—H6A	0.9900	C15—C16	1.388 (4)
C6—H6B	0.9900	C15—H15	0.9500
C7—N2	1.515 (3)	C16—C17	1.389 (4)
C7—C8	1.520 (4)	C16—N5	1.444 (3)
C7—H7A	0.9900	C17—C18	1.375 (4)
C7—H7B	0.9900	C17—H17	0.9500
C8—N3	1.461 (3)	C18—H18	0.9500
C8—H8A	0.9900	N5—O1	1.233 (3)
C8—H8B	0.9900	N5—O2	1.234 (3)
			
N1—C1—C2	111.1 (2)	N4—C10—C9	111.9 (2)
N1—C1—H1A	109.4	N4—C10—H10A	109.2
C2—C1—H1A	109.4	C9—C10—H10A	109.2
N1—C1—H1B	109.4	N4—C10—H10B	109.2
C2—C1—H1B	109.4	C9—C10—H10B	109.2
H1A—C1—H1B	108.0	H10A—C10—H10B	107.9
N2—C2—C1	108.0 (2)	N3—C11—C12	111.0 (2)
N2—C2—H2A	110.1	N3—C11—H11A	109.4
C1—C2—H2A	110.1	C12—C11—H11A	109.4
N2—C2—H2B	110.1	N3—C11—H11B	109.4
C1—C2—H2B	110.1	C12—C11—H11B	109.4
H2A—C2—H2B	108.4	H11A—C11—H11B	108.0
N1—C3—C4	110.9 (2)	N4—C12—C11	110.6 (2)
N1—C3—H3A	109.5	N4—C12—H12A	109.5
C4—C3—H3A	109.5	C11—C12—H12A	109.5
N1—C3—H3B	109.5	N4—C12—H12B	109.5
C4—C3—H3B	109.5	C11—C12—H12B	109.5
H3A—C3—H3B	108.0	H12A—C12—H12B	108.1
N2—C4—C3	107.7 (2)	N4—C13—C14	121.1 (2)
N2—C4—H4A	110.2	N4—C13—C18	121.1 (2)
C3—C4—H4A	110.2	C14—C13—C18	117.7 (2)
N2—C4—H4B	110.2	C15—C14—C13	121.2 (2)
C3—C4—H4B	110.2	C15—C14—H14	119.4
H4A—C4—H4B	108.5	C13—C14—H14	119.4
N1—C5—C6	110.9 (2)	C14—C15—C16	119.6 (2)
N1—C5—H5A	109.5	C14—C15—H15	120.2
C6—C5—H5A	109.5	C16—C15—H15	120.2
N1—C5—H5B	109.5	C15—C16—C17	120.6 (2)
C6—C5—H5B	109.5	C15—C16—N5	120.1 (2)
H5A—C5—H5B	108.0	C17—C16—N5	119.3 (2)
N2—C6—C5	108.3 (2)	C18—C17—C16	120.2 (2)
N2—C6—H6A	110.0	C18—C17—H17	119.9
C5—C6—H6A	110.0	C16—C17—H17	119.9
N2—C6—H6B	110.0	C17—C18—C13	120.5 (2)
C5—C6—H6B	110.0	C17—C18—H18	119.8
H6A—C6—H6B	108.4	C13—C18—H18	119.8
N2—C7—C8	116.1 (2)	C3—N1—C1	108.4 (2)
N2—C7—H7A	108.3	C3—N1—C5	108.7 (2)
C8—C7—H7A	108.3	C1—N1—C5	107.6 (2)
N2—C7—H7B	108.3	C2—N2—C7	111.5 (2)
C8—C7—H7B	108.3	C2—N2—C6	108.47 (19)
H7A—C7—H7B	107.4	C7—N2—C6	107.45 (19)
N3—C8—C7	115.3 (2)	C2—N2—C4	108.49 (19)
N3—C8—H8A	108.4	C7—N2—C4	112.71 (19)
C7—C8—H8A	108.4	C6—N2—C4	108.07 (19)
N3—C8—H8B	108.4	C8—N3—C9	110.5 (2)
C7—C8—H8B	108.4	C8—N3—C11	111.7 (2)
H8A—C8—H8B	107.5	C9—N3—C11	108.1 (2)
N3—C9—C10	110.5 (2)	C13—N4—C10	119.7 (2)
N3—C9—H9A	109.6	C13—N4—C12	119.2 (2)
C10—C9—H9A	109.6	C10—N4—C12	112.1 (2)
N3—C9—H9B	109.6	O1—N5—O2	122.9 (2)
C10—C9—H9B	109.6	O1—N5—C16	118.7 (2)
H9A—C9—H9B	108.1	O2—N5—C16	118.4 (2)
			
N1—C1—C2—N2	17.9 (3)	C8—C7—N2—C6	168.3 (2)
N1—C3—C4—N2	17.4 (3)	C8—C7—N2—C4	−72.8 (3)
N1—C5—C6—N2	16.5 (3)	C5—C6—N2—C2	−67.9 (3)
N2—C7—C8—N3	65.3 (3)	C5—C6—N2—C7	171.4 (2)
N3—C9—C10—N4	56.7 (3)	C5—C6—N2—C4	49.5 (3)
N3—C11—C12—N4	−57.3 (3)	C3—C4—N2—C2	48.9 (3)
N4—C13—C14—C15	−177.3 (2)	C3—C4—N2—C7	172.9 (2)
C18—C13—C14—C15	0.2 (4)	C3—C4—N2—C6	−68.5 (2)
C13—C14—C15—C16	1.1 (4)	C7—C8—N3—C9	−171.5 (2)
C14—C15—C16—C17	−1.2 (4)	C7—C8—N3—C11	68.1 (3)
C14—C15—C16—N5	−178.7 (2)	C10—C9—N3—C8	177.2 (2)
C15—C16—C17—C18	0.0 (4)	C10—C9—N3—C11	−60.3 (3)
N5—C16—C17—C18	177.5 (2)	C12—C11—N3—C8	−177.0 (2)
C16—C17—C18—C13	1.3 (4)	C12—C11—N3—C9	61.3 (3)
N4—C13—C18—C17	176.0 (2)	C14—C13—N4—C10	−32.4 (4)
C14—C13—C18—C17	−1.4 (4)	C18—C13—N4—C10	150.2 (2)
C4—C3—N1—C1	−68.6 (3)	C14—C13—N4—C12	−176.9 (2)
C4—C3—N1—C5	48.1 (3)	C18—C13—N4—C12	5.8 (4)
C2—C1—N1—C3	47.9 (3)	C9—C10—N4—C13	161.0 (2)
C2—C1—N1—C5	−69.5 (3)	C9—C10—N4—C12	−52.2 (3)
C6—C5—N1—C3	−68.3 (3)	C11—C12—N4—C13	−161.0 (2)
C6—C5—N1—C1	48.9 (3)	C11—C12—N4—C10	52.0 (3)
C1—C2—N2—C7	166.4 (2)	C15—C16—N5—O1	15.9 (3)
C1—C2—N2—C6	48.3 (3)	C17—C16—N5—O1	−161.6 (2)
C1—C2—N2—C4	−68.9 (3)	C15—C16—N5—O2	−165.1 (2)
C8—C7—N2—C2	49.5 (3)	C17—C16—N5—O2	17.4 (3)

### Hydrogen-bond geometry (Å, º)

**Table:**

*D*—H···*A*	*D*—H	H···*A*	*D*···*A*	*D*—H···*A*
C2—H2*B*···O1^i^	0.99	2.52	3.051 (3)	113
C5—H5*A*···O2^ii^	0.99	2.47	3.414 (3)	160
C6—H6*B*···O1^ii^	0.99	2.59	3.141 (3)	116
C14—H14···I1^i^	0.95	3.02	3.914 (3)	158
C17—H17···O2^iii^	0.95	2.48	3.412 (3)	168
C18—H18···N1^iv^	0.95	2.45	3.384 (3)	166

Symmetry codes: (i) −*x*+1, −*y*+1, −*z*+1; (ii) *x*+1, −*y*+1/2, *z*−1/2; (iii) −*x*, −*y*, −*z*+1; (iv) −*x*+1, *y*−1/2, −*z*+1/2.
